# Compression‐Tension‐Asymmetry and Stiffness Nonlinearity of Collagen‐Matrigel Composite Hydrogels

**DOI:** 10.1002/adhm.202503052

**Published:** 2025-12-05

**Authors:** David Böhringer, Jan Hinrichsen, Radik Gataulin, Sandra Wiedenmann, Marina Spörrer, Selda Sherifova, Paul Steinmann, Gerhard A. Holzapfel, Ben Fabry, Silvia Budday

**Affiliations:** ^1^ Department of Physics Friedrich‐Alexander‐Universität Erlangen‐Nürnberg 91058 Erlangen Germany; ^2^ Institute of Continuum Mechanics and Biomechanics Friedrich‐Alexander‐Universität Erlangen‐Nürnberg 91058 Erlangen Germany; ^3^ Institute of Biomechanics Graz University of Technology 8010 Graz Austria; ^4^ Mines Saint‐Étienne INSERM U1059 SAINBIOSE Saint‐Étienne F‐42023 France; ^5^ Institute of Applied Mechanics Friedrich‐Alexander‐Universität Erlangen‐Nürnberg 91058 Erlangen Germany; ^6^ Department of Structural Engineering Norwegian University of Science and Technology (NTNU) Trondheim 7491 Norway

**Keywords:** biopolymer, finite element method, mechanical testing, ogden model, parameter identification

## Abstract

Collagen type I hydrogels, which self‐assemble into 3D fiber networks, are commonly used for cell culture and tissue engineering applications. Collagen hydrogels replicate the nonlinear stress–strain relationship of collagenous tissue under extension. However, they buckle and soften under compression, whereas natural tissue exhibits significant stiffening due to the presence of cells and other matrix components. To more closely mimic the mechanical properties of natural tissue, varying concentrations of the basement membrane extract Matrigel are added to collagen. The stress–strain relationship of the resulting composite hydrogels is then analyzed under compression, tension, and shear. It is found that the addition of Matrigel increases the stiffness and reduces the compression‐tension asymmetry. This can be explained by a reduced degree of freedom for collagen fiber buckling due to the constraints imposed by the surrounding fine‐meshed Matrigel network. Consistent with this explanation, it is found that the collapse of composite hydrogels under uniaxial strain decreases with increasing concentration of Matrigel and other filler materials, such as alginate. Taken together, by adjusting the ratio of Matrigel to collagen, the mechanical compression‐tension asymmetry and nonlinearity of composite hydrogels can be tuned to more closely mimic natural tissue and tailor cell behavior.

## Graphical abstract

Self‐assembled collagen hydrogel matrices are widely used in tissue engineering applications. These matrices stiffen and contract laterally under tension due to fiber alignment and soften and collapse under compression due to fiber buckling. It is demonstrated that filler materials, such as Matrigel, linearize the mechanical behavior of collagen hydrogels by reducing their lateral contraction under tension and collapse under compression.

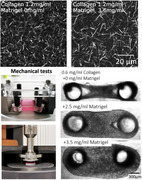

## 1 Introduction

Collagen hydrogels, generated from acid‐solubilized monomers that assemble into fiber networks, are commonly used as a 3D matrix for cell studies^[^ ^1^, ^2^, ^3^, ^4^, ^5^ ^]^ and in tissue engineering.^[^ ^6^, ^7^, ^8^, ^9^ ^]^ Since collagen is a highly abundant protein in the connective tissue of most organs, self‐assembled collagen hydrogels are thought to mimic the natural extracellular environment of cells at least to some degree.^[^ ^10^, ^11^ ^]^

The mechanical properties of the extracellular matrix, in particular its strain‐dependent elastic properties, are a major determinant of cell behavior including cell differentiation^[^ ^4^, ^12^ ^]^ and cell migration.^[^ ^1^, ^2^, ^3^, ^13^, ^14^ ^]^ Under small strains up to 3%, the elasticity (stiffness) of fiber networks, including collagen fiber networks, is typically characterized by a strain‐independent (constant) shear modulus, while for higher strains above 3–10%, the stiffness of fiber networks increases nonlinearly.^[^ ^15^, ^16^ ^]^ Moreover, self‐assembled collagen hydrogels respond not only to the strain magnitude but also to the strain direction, and hence show a pronounced mechanical asymmetry ‐ their stiffness increases under tension but decreases under compression. This behavior can be explained by the strong nonlinear stiffening of collagen fibers under tension and their buckling and softening under compression.^[^ ^11^, ^12^, ^15^, ^16^, ^17^, ^18^ ^]^

The strain stiffening of collagen hydrogels under tension is qualitatively similar to that observed in natural collagenous tissue.^[^ ^10^, ^11^, ^16^ ^]^ The strain softening of collagen hydrogels under compression, however, is not observed in natural tissue, which rather shows significant stiffening. This stiffening under compression of natural tissue can be attributed to the presence of volume‐preserving components, such as cells or other matrix components.^[^ ^11^ ^]^

Although the softening of collagen‐based hydrogels under compression may seem non‐physiologic, it can in fact be highly beneficial in several situations. In particular, the pronounced collapse of self‐assembled collagen hydrogels under compression allows for the formation of dense artificial tissues. When cells are mixed into a collagen gel, they can adhere to the collagen fibers after polymerization, spread out, contract, and compress and densify the collagen network.^[^ ^19^, ^20^, ^21^ ^]^

Depending on the geometry of the dish containing the collagen gel, the ability of the collagen fibers to adhere to the surface, and the magnitude of cell‐generated contractile forces, the collagen gel will aggregate into a compact structure. If, for example, the dish is a non‐adhesive U‐shaped microwell, a spheroid is formed.^[^ ^22^, ^23^ ^]^ If the dish contains two or more pillars, a tissue of aligned collagen fibers and cells forms between these pillars.^[^ ^24^, ^25^, ^26^ ^]^ Such micro‐tissues can then be used to study the functional properties of, for example, skeletal‐ or cardiac muscle tissue.

The alignment of collagen fibers under tensile forces is also exploited in tumor models, where cells in a collagen matrix generate contractile forces that produce highly aligned collagen strands that facilitate cell communication and radial‐directed cell invasion into the surrounding matrix.^[^ ^27^, ^28^, ^29^, ^30^, ^31^ ^]^

For replicating the mechanical properties of real tissue, however, the strong collapse of self‐assembled collagen hydrogels under compressive load remains a limitation.

In this study, we hypothesize that the basement membrane extract Matrigel, which contains various matrix proteins such as laminin and collagen IV^[^ ^32^ ^]^ and shows good cell compatibility,^[^ ^33^, ^34^, ^35^ ^]^ may serve as a volume‐preserving component in Matrigel‐collagen composite hydrogels. This could enable tuning of the mechanical behavior, while maintaining high cell compatibility. We incorporate varying concentrations of Matrigel to collagen type I hydrogels and characterize the nonlinear mechanical behavior using inverse parameter identification. Specifically, we fit a nonlinear finite element model based on the hyperelastic one‐term Ogden model to the experimentally measured stress‐strain data of Matrigel‐collagen composites under compression and tension. The Ogden model is especially well‐suited to capture the compression‐tension asymmetry typical of collagen‐based hydrogels.^[^ ^36^, ^37^ ^]^ Our results demonstrate that the addition of Matrigel allows us to tune the mechanical compression‐tension asymmetry and the nonlinearity of the composite hydrogels.

## 2 Experimental Section

### 2.1 Hydrogel Preparation

#### 2.1.1 Collagen‐Matrigel gels

Collagen type I hydrogels were prepared from acid‐dissolved rat tail (R) and bovine skin (G1) collagen (Matrix Bioscience, Mörlenbach), mixed on ice at a mass ratio of 1:2 and dissolved in a solution of 1 vol part NaHCO_3_, 1 vol. part 10 ×  Dulbecco's Modified Eagle Medium (10xDMEM, Seraglob, Schaffhausen) and 8 vol. parts H_2_O. For the composite hydrogels, the basement membrane extract Matrigel (Corning, New York) was added at different concentrations to the collagen solution prior to polymerization. The pH value of the solution was adjusted to 9 using NaOH, after which the hydrogel is polymerized for 1 h at 37°C.

Pure collagen hydrogels was used at concentrations of 0.6 (C06), 1.2 (C12), and 2.4 mgml^−1^ (C24), and pure Matrigel hydrogels at a concentration of 10 mgml^−1^. In addition, another basement membrane extract, Geltrex, was used at the stock concentration of 14.6 mgml^−1^ (Thermo Fisher, Waltham). Collagen‐Matrigel composite hydrogels are mixed at concentrations of 1.2 mgml^−1^ collagen and 1.2 (C12M12), 2.4 (C12M24), or 3.6 mgml^−1^ (C12M36) Matrigel.

For the mechanical tests with collagen and Matrigel mixtures, a single batch of Matrigel and collagen was used, as both materials are known to display batch‐to‐batch variability. The muscle microtissue experiments and the uniaxial stretch experiments with different matrices were conducted with different collagen and Matrigel batches.

#### 2.1.2 ColMA‐HASH Gels

Thiol‐modified hyaluronic acid (HA‐SH) was synthesized as described in Ref. [38]. For hydrogel preparation, methacrylated collagen (ColMA, CellSystems, Troisdorf) was solubilized in 20 mM acetic acid at 4°C. HA‐SH (MW 538 kDa) and the photoinitiator LAP (CellSystems, Troisdorf) were each dissolved separately in HEPES buffer (154 mM) at room temperature on a shaker. The solutions were then combined at 4°C to a final concentrations of 1.5 mgml^−1^ ColMA, 1.0 mgml^−1^ HA‐SH, and 0.8 mgml^−1^ LAP. The pH was adjusted to seven with NaOH and the solution was kept on ice for 45 min until no visible bubbles remain. Gels were cross‐linked with blue light (405 nm) for 2 min at a power density of 2.4 mWcm^−2^.

#### 2.1.3 Collagen‐Alginate Gels

Sodium alginate (Vivapharm PH176, JRS Pharma, Rosenberg) was dissolved at 2% (w/v) in serum‐free DMEM with overnight stirring at room temperature. The solution was equilibrated for 6 h at 37°C, 5% CO_2_, and 95% RH, then mixed on ice with collagen to a final concentration of 1.0 mgml^−1^ alginate and 1.5 mgml^−1^ collagen.

### 2.2 Collagen Microstructure

Collagen type I hydrogels and collagen‐Matrigel composite hydrogels (600 µl hydrogel volume) were cast into 3D printed plastic rings (12 mm diameter, and 5.4 mm hydrogel height, Shapeways, New York) and polymerized for 1 h at room temperature or at 37°C with 95% humidity and 5% CO2. Afterwards, the samples were covered with 3 ml phosphate buffered saline solution (PBS) and transferred to an upright confocal laser scanning microscope (Leica SP5, Leica, Wetzlar) equipped with a 20x dip‐in objective (NA 1.0, Leica, Wetzlar). The microstructure of the hydrogels was imaged using confocal reflection microscopy, whereby a total volume of 82× 82 ×81 µm (voxelsize 161× 161 ×593 nm) was imaged.

The pore diameter of collagen networks was measured as previously described.^[^ ^39^, ^40^ ^]^ Briefly, confocal reflection microscopy images of the collagen network are binarized using Li thresholding^[^ ^41^ ^]^ and subsequently skeletonized, reducing collagen fibers to their centerlines. The pores were then identified as local maxima in the distance transform map of the skeletonized images. The measured 2D pore diameters were converted to effective 3D pore diameters using a stereological correction factor of 1.36 that had been empirically determined for collagen gels.^[^ ^40^ ^]^ To account for reduced reflectivity of vertically oriented fibers during confocal reflection imaging, a blind‐spot correction factor of 0.79 was applied, which had also been empirically determined for collagen gels in a previous study.^[^ ^42^ ^]^

### 2.3 Cell Culture

C2C12 skeletal muscle cells were cultured at 37°C with 95% humidity and 5% CO2 using DMEM medium (4.5 mgml^−1^ glucose, Thermo Fisher, Waltham) supplemented with 15% FCS (Thermo Fisher, Waltham), 1% penicillin streptomycin (Thermo Fisher, Waltham), 2mM non‐essential aminoacids (MEM NEAA 100x, Thermo Fisher, Waltham), 1 mM sodium pyruvate (100x, Thermo Fisher, Waltham) and 1mM GlutaMaxx (100x, Thermo Fisher, Waltham). The differentiation medium used after microtissue assembly consisted of DMEM medium supplemented with 2% horse serum (Thermo Fisher, Waltham), 1% penicillin streptomycin, 2mM non‐essential aminoacids, 1 mM sodium pyruvate, 1 mM GlutaMax and insulin transferrin‐selenium (ITS, Thermo Fisher, Waltham) in a dilution of 1:200.

### 2.4 Muscle Microtissue

Contractile muscle tissues were prepared in custom‐made polydimethylsiloxane (PDMS) culture devices around two flexible pillars similar as described in Ref. [25, 26]. The PDMS devices were fabricated using a crosslinker‐to‐base ratio of 1:22 (Sylgard 184, Sigma‐Aldrich, St. Louis) and cured for 24 h at 65 °C. The PDMS devices were then sterilized under UV light for 5 min. To prevent cell adhesion to the walls, the devices were functionalized with 1% Pluronic acid solution (Sigma‐Aldrich, St. Louis) for 1 h at room temperature, after which the Pluronic acid was removed.

To set the muscle tissue height, a base layer of extracellular matrix (ECM) was first added to each chamber. The ECM consists of 0.6 mgml^−1^ collagen I solution (Matrix Bioscience, Mörlenbach) and Matrigel (Corning, New York) at different concentrations prepared on ice to prevent premature polymerization. A volume of 6 µL ECM was pipetted into each chamber and centrifuged (750 rpm, 1 min) to ensure even distribution, followed by 1 h incubation at 37°C for gelation. Here, the PDMS culture devices were placed on a layer of ice to slow down the gelation process. Afterwards, a top layer consisting of 7500 C2C12 cells mixed in 6 µL of ECM layer added per chamber. After 1 h of incubation, 1 ml culture medium was added. After 24 h, when compact tissues self‐organized around the micropillars, the medium was replaced with differentiation medium (see Section 2.3) and renewed every second day.

After six days, the contractility of microtissues was measured under electrical stimulation as previously described.^[^ ^25^, ^26^, ^43^ ^]^ Here, tissues were electrically stimulated using a custom‐build pacing device with bipolar pulses at 0.5 Hz, 16.8 V, 2 ms pulse width. The contractility of the microtissues was then determined from the pillar deflection and the tissue height measured dynamically by using brightfield microscopy (Leica DMI6000, Wetzlar). Pillar deflections were quantified using ClickPoints software,^[^ ^44^ ^]^ and contractile forces were calculated using Euler–Bernoulli beam theory as described previously.^[^ ^25^, ^26^ ^]^

### 2.5 Mechanical Testing

#### 2.5.1 Rheometer Experiments

**Figure 1:**
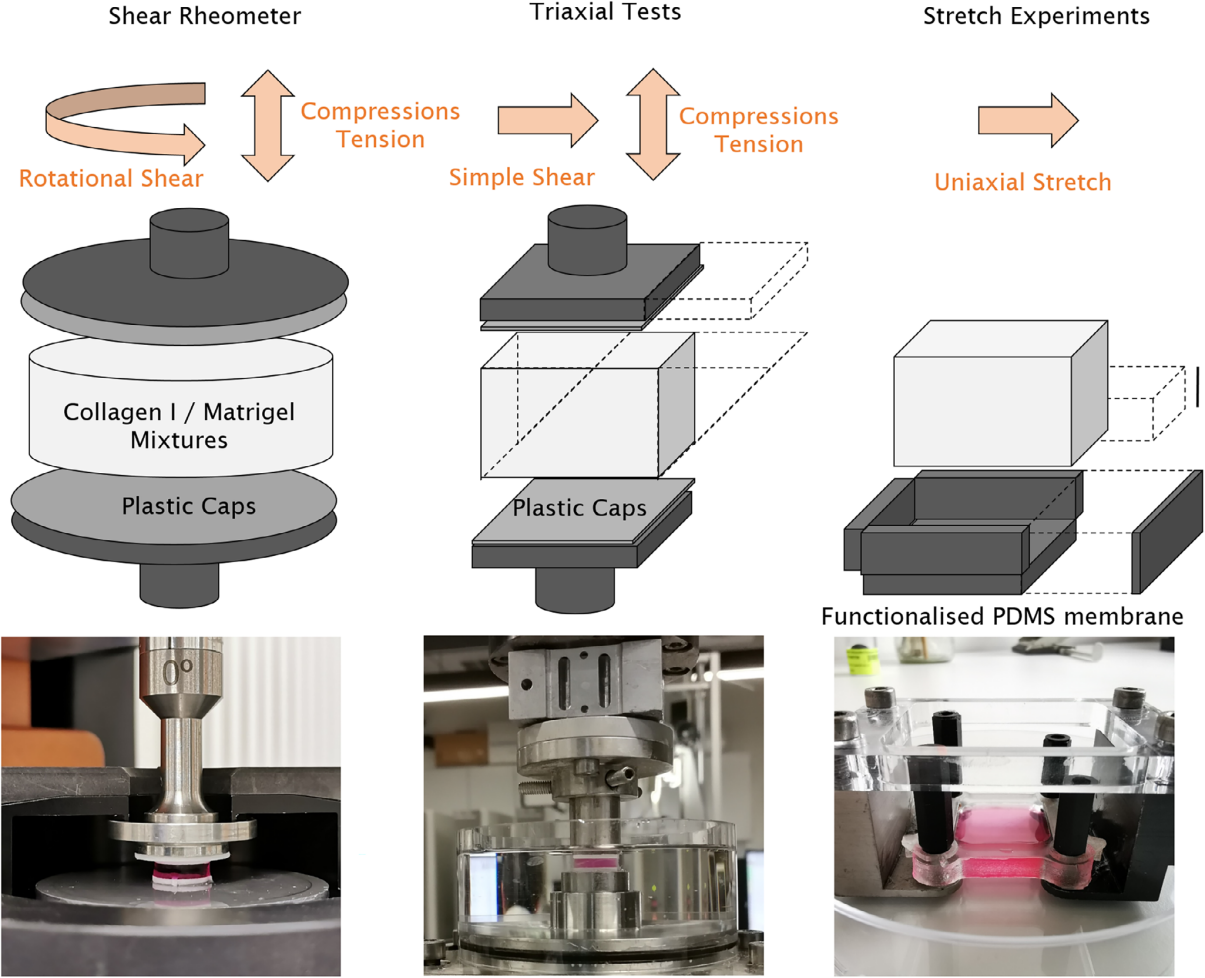
Experimental setup. Composite hydrogels were prepared at different ratios of acid‐dissolved collagen (type I) and basement membrane extract Matrigel. The mechanical behavior of the hydrogels was characterized under finite deformations using different experimental setups: A shear rheometer (left), a triaxial testing device (middle), and an uniaxial stretch device (right).

Hydrogel mechanics were first tested using a Discovery HR‐3 rheometer (TA Instruments, Milford; see **Figure** **1**) in combination with a plate‐plate geometry (20 mm diameter, TA Instruments, Milford). To ensure proper adhesion of the hydrogels to the rheometer plates, porous plastic caps (20 mm diameter on the rheometer side with a 12 mm diameter plateau on the hydrogel side, Shapeways, New York) were glued to the upper and lower rheometer plates. Cylindrically shaped hydrogels (12 mm diameter, 3 mm height) were polymerized for 1 h between the rheometer plates. During polymerization, the plates were held at 37°C, and a water‐filled solvent trap (TA Instruments, Milford) prevents evaporation. Afterwards, the following protocol was used for mechanical testing. At least three samples were tested per condition (**Table** **1**).

**Table 1:** Sequential test protocol (shear rheometer).

1	**Frequency sweep**	Sinusoidal shear at a strain of γ = 0.01 at frequencies of 0.02–1 Hz
2	**Torsional shear**	3 cycles at a strain of γ = 0.2 at a constant rate of 0.01s^−1^
3	**Stress relaxation**	Hold torsional strain of γ = 0.2 for 300 s then reduce strain to γ = 0 and hold for 30 s
4	**Torsional shear**	3 cycles at a strain of γ = 0.2 at a constant rate of 0.01s^−1^
5	**Stress relaxation**	Hold torsional strain of γ = 0.2 for 300 s then reduce strain to γ = 0 and hold for 30 s
6	**Compression**	3 cycles at a strain of ϵ = 0.1 at a constant rate of 0.01s^−1^
7	**Stress relaxation**	Hold compressive strain of ϵ = 0.1 for 300 s then reduce strain to ϵ = 0 and hold for 30 s
8	**Tension**	3 cycles at a strain of ϵ = 0.1 at a constant rate of 0.01s^−1^
9	**Stress relaxation**	Hold tensile strain of ϵ = 0.1 for 300 s then reduce strain to ϵ = 0 and hold for 30 s

#### 2.5.2 Triaxial Experiments

In addition to measurements in a plate‐plate rheometer, mechanical measurements were also performed using a triaxial testing device as described in Ref. [45] (Figure 1). The hydrogels were polymerized for 1 h at approx. 37°C and 95% humidity between two rectangular porous plastic caps (10 mm×10 mm, Shapeways, New York, gap width 2.4‐3.0 mm) that were glued to the upper and lower plates to ensure proper sample adhesion. A custom‐made mold was used to prevent leakage on the sides of the geometry. After polymerization, the filling mold was removed and the measurement chamber was flooded with cell culture medium (DMEM, low glucose, Thermo Fisher, Waltham). Mechanical tests were conducted following the protocol specified in (**Table** **2**):

**Table 2:** Sequential test protocol (triaxial).

1	**Simple shear (x‐direction)**	3 cycles at a strain of γ = 0.2 at a constant rate of 0.01s^−1^
2	**Stress relaxation**	Hold shear strain of γ = 0.2 for 300 s then reduce strain to γ = 0 and hold for 30 s
3	**Simple shear (y‐direction)**	3 cycles at a strain of γ = 0.2 at a constant rate of 0.01s^−1^
4	**Stress relaxation**	Hold shear strain of γ = 0.2 for 300 s then reduce strain to γ = 0
5	**Compression**	3 cycles at a strain of ϵ = 0.1 at a constant rate of 0.01s^−1^
6	**Stress relaxation**	Hold compressive strain of ϵ = 0.1 for 300 s then reduce strain to ϵ = 0 and hold for 30 s
7	**Tension**	3 cycles at a strain of ϵ = 0.1 at a constant rate of 0.01s^−1^
8	**Stress relaxation**	Hold tensile strain of ϵ = 0.1 for 300 s then reduce strain to ϵ = 0 and hold for 30 s

#### 2.5.3 Uniaxial Stretch Experiments

Custom‐made PDMS stretch membranes (Sylgard184, Sigma–Aldrich, St. Louis; see Figure 1) were prepared as previously described in Ref. [46]. Here, a crosslinker‐to‐base ratio of 1:14 was used. PDMS membranes were cured for 24 h at 65°C. The inner surface of the membranes (area of ≈4 cm^2^) was treated with 0.5 mgml^−1^ sulfo‐SANPAH (5 min activation with UV light, Thermo Fisher, Waltham) for better hydrogel attachment. To ensure a flat geometry, the membranes were uniaxially pre‐stretched to 5% strain prior to hydrogel addition, using a custom‐built stepper motor stretching device.^[^ ^47^ ^]^ Next, the hydrogels were prepared on ice and mixed with 4 µm diameter microbeads (5 µl per 1 ml hydrogel, PSI‐4.0COOH, Kisker, Steinfurt). A volume of 700 µl of the mixture was transferred to the stretch membranes and pre‐polymerized for 20 min in a cell culture incubator (37°C with 95% humidity and 5% CO_2_). Additional microbeads (5 µl) were then pipetted onto the gel surface, and the gel was fully polymerized in the incubator for another 40 min. Afterwards, 1 ml cell culture medium (DMEM, low glucose, Thermo Fisher, Waltham) was added, and the hydrogels were transferred to a fluorescence microscope (Leica DMI6000 CS with a 20x Leica HCX PL Fluotator objective, Leica, Wetzlar) equipped with a custom‐made stepper‐motor driven stretching device^[^ ^47^ ^]^ mounted on the microscope stage. The hydrogels were then uniaxially stretched in approx. 1% steps, each step lasting for 30 s. For estimating the apparent Poisson's ratio, the gel thickness was measured at each step by focusing on the top and bottom surfaces, using the embedded microbeads as fiducial markers. At least three samples were tested per condition.

### 2.6 Pre‐Processing

**Figure 2:**
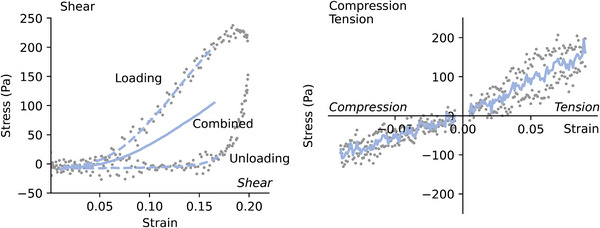
Pre‐processing of the experimental data. The data points from the initial loading and unloading curve (grey dots) of the shear rheological (left) and the compressive and tensile tests (right) were interpolated (blue dashed lines) and averaged (blue solid lines) to extract the hyperelastic response (combined curve). The combined curves were further averaged over all individual samples for each condition (see Figure 5).

The materials tested in this study are viscoelastic and showed a pronounced hysteresis during cyclic loading (see **Figure** **2**). Here, the focus of study is on time‐independent, hyperelastic effects, i.e., nonlinearity and compression‐tension asymmetry. To remove the dynamic responses from the measurements, the loading and unloading curves of the first compression and tension cycle (Figure 2 left) were averaged.

For torsional tests using the shear rheometer, the torque *T* was measured and cthe torsional shear stress was calculated as τ = 2*T*/π*r* ^3^, with plate radius *r*. The shear strain was calculated as γ = φ*r*/*h*, with torsional angle φ and sample height *h*. For the compression and tension measurements, the strain was computed as ϵ = Δ*x*/*h*, with axial displacement Δ*x*. The nominal stress was calculated from the axial force *f*_*ax*_ as *P*_*ax*_ = *f*_*ax*_/π*r* ^2^. For the triaxial testing device, the shear stress σ was calculated from the shear force *f*_*sh*_ and the undeformed surface area A as σ = *f*_*sh*_/*A*.

For each loading cycle, the raw stress and strain data was interpolated to obtain stress values at evenly spaced intervals of strain with a spacing of 0.001. Subsequently, loading and unloading curves were averaged. To minimize artifacts from acceleration and deceleration, data near the strain inflection points (up to 0.01 for shear rheometer measurements and up to 0.03 for triaxial testing device measurements) were discarded. The resulting curves were then averaged over all samples for each hydrogel mixture, as shown in Figure 5.

**Figure 3:**
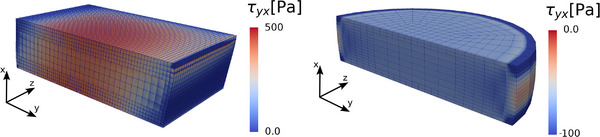
Inhomogeneous sample deformations. Deformed geometries from finite element (FE) simulations for the cuboid shaped samples (triaxial testing device) under simple shear (left) and the cylindrical shaped samples (rheometer) under compression (right). Both visualizations show a sectional view of the specimens. The bulging on the sides as well as the local stress concentrations (Kirchhoff stress **τ**) indicate an inhomogeneous deformation state.

**Figure 4:**
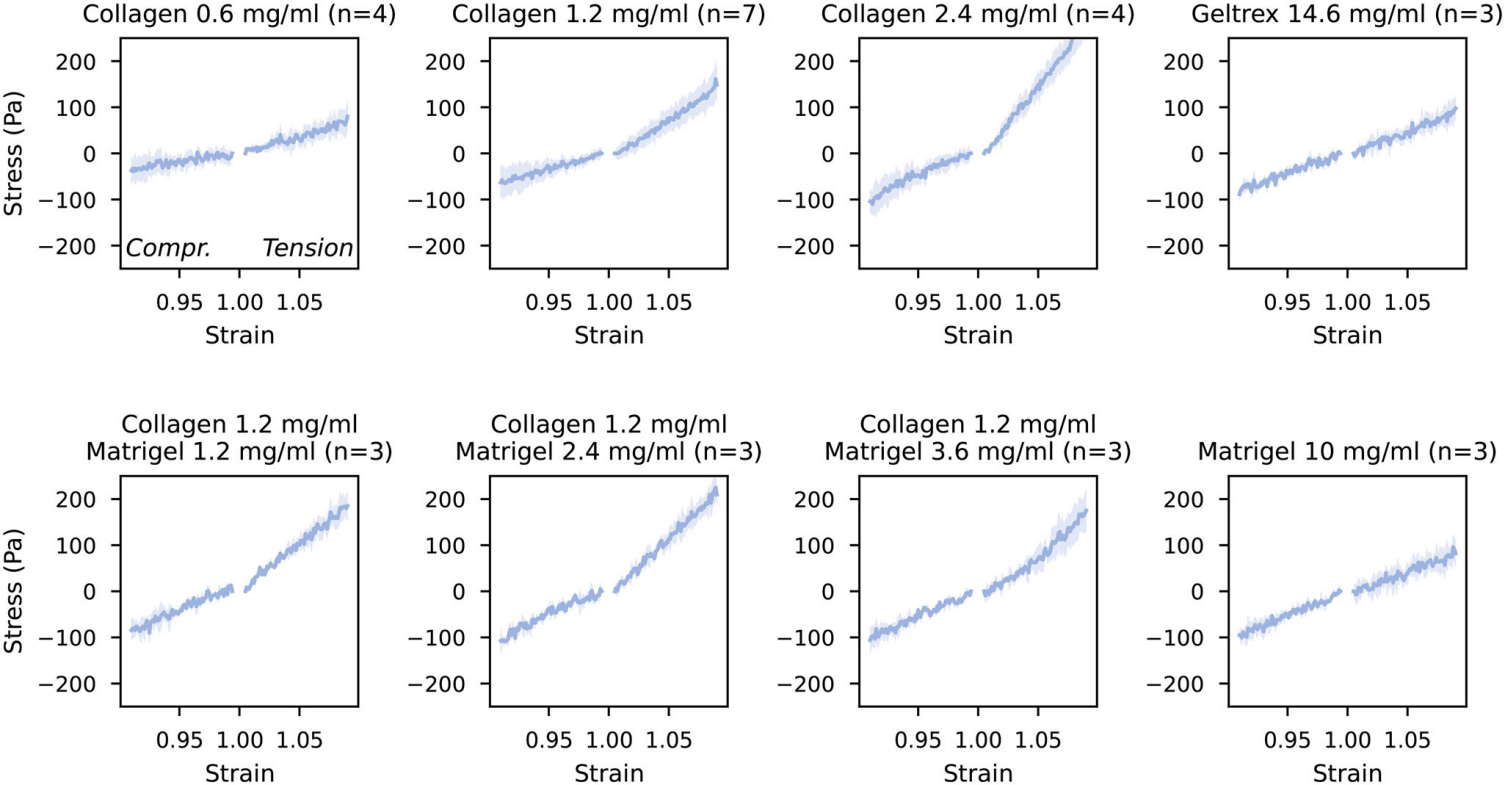
Compression‐tension experiments, Stress–strain relationship under compressive and tensile loading obtained from the rheometer experiments. Lines and shaded areas show the mean value and standard deviation over individual samples per hydrogel formulation consisting of Matrigel, Collagen and Geltrex at different concentrations. Curves show the average of the first loading and unloading cycle. n indicates the number of individual samples.

**Figure 5:**
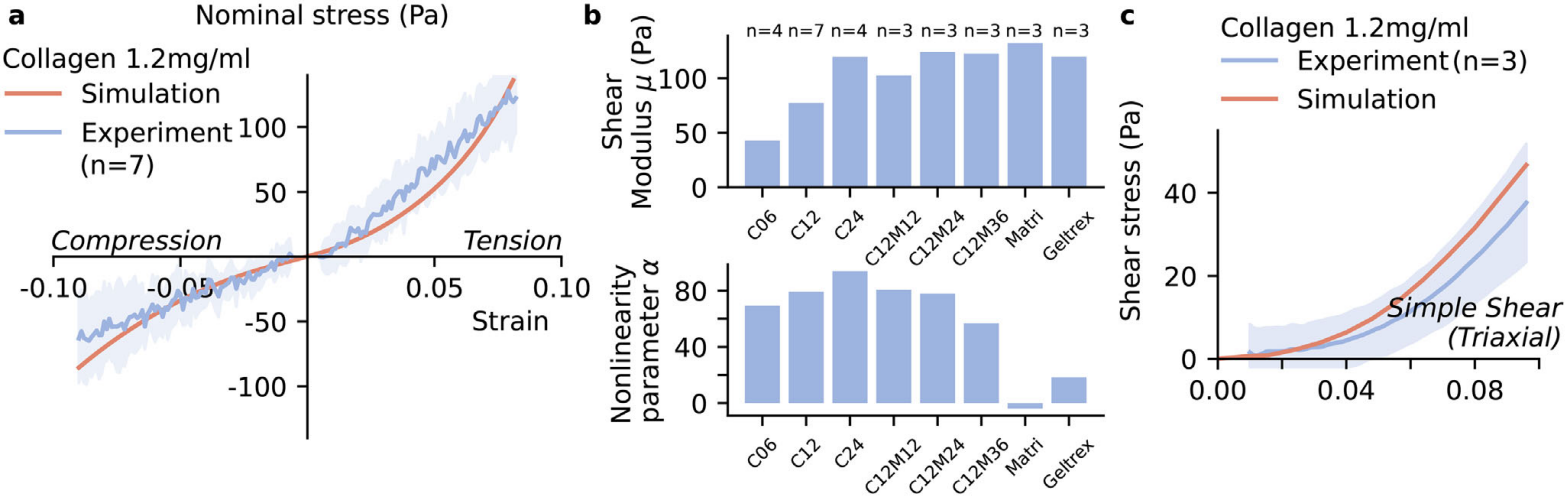
Tuning the stiffness and compression tension asymmetry in Matrigel collagen blends. a) Stress–strain relationship obtained from experimental rheometer data (blue) and from the Ogden model fit (orange) for the initial cycle of the compression tension experiments for 1.2 mgml^−1^ collagen gels. Line and shaded area show mean±sd for seven individual samples. b) The shear modulus µ (top) and the nonlinearity parameter α (bottom) are obtained from the fitted hyperelastic Ogden model for different hydrogel mixtures. Here, the Ogden model is fitted to the mean response over all samples per hydrogel mixture, where n is the number of individual samples. Hydrogels are collagen gels at a concentration of 0.6 mgml^−1^ (C06), 1.2 mgml^−1^ (C12), 2.4 mgml^−1^ (C24), Geltrex at a concentration of 14.6 mgml^−1^, Matrigel at a concentration of 10 mgml^−1^, and Matrigel‐collagen blends all using 1.2 mgml^−1^ collagen and 1.2 mgml^−1^ Matrigel (C12M12), 2.4 mgml^−1^ Matrigel (C12M24), or 3.6 mgml^−1^ Matrigel (C12M36) **c)** A simple shear experiment of 1.2 mgml^−1^ collagen gels is performed using a different experimental setup (triaxial tests). The shear stress response is then simulated using parameters obtained from fitting the rheometer compression–tension experiments of 1.2 mgml^−1^ collagen gels (data shown in a). The blue line and shaded area show mean±sd of three individual samples.

### 2.7 Modeling and Parameter Identification

To model the mechanical response of the materials, nonlinear continuum mechanics and finite element simulations (implemented in deal.ii^[^ ^48^ ^]^) were used. The aim was to approximate the experimental data by a computational model, which was then used to identify material parameters describing the stiffness, nonlinearity, and compression‐tension asymmetry.

#### 2.7.1 Constitutive Modeling

For the constitutive equation describing the relationship between stress and strain, the hyperelastic one‐term Ogden model was used,^[^ ^49^ ^]^ which captures the compression‐tension asymmetry of collagen.^[^ ^45^ ^]^ For finite element simulations, points in the material configuration **X** are mapped to the deformed configuration **x** by the deformation map φ: **X** → **x**. The principal stretches λ can be computed from the square root of the principal values of the left Cauchy‐Green tensor **b** = **F** **F** ^*T*^, where the deformation gradient **F** is defined as Grad φ(**X**).^[^ ^50^ ^]^ For hyperelastic materials, the existence of a strain‐energy density Ψ can be postulated that depends only on the deformation, which is characterized by **F** or its derived strain tensors. In line with the observed collagen structure (Figure S8– S10, Supporting Information), isotropic material behavior was assumed at macroscopic scales (>50 µm,^[^ ^42^ ^]^). The Cauchy stress can be written as

(1) $$\sigma =J^{-1}\frac{\partial \Psi }{\partial F}F^{T}$$

where *J* = det**F** denotes the volume ratio.^[^ ^51^ ^]^ To characterize the different material behavior in bulk and shear independently, the strain‐energy density was splited into an isochoric and a volumetric part

(2) $$\Psi =\Psi_{\mathrm{iso}}+\Psi_{\mathrm{vol}}$$

The isochoric part is defined as

(3) $$\Psi_{\mathrm{iso}}=\frac{2\mu }{\alpha^{2}}\left[{\bar{\lambda }}_{1}^{\alpha }+{\bar{\lambda }}_{2}^{\alpha }+{\bar{\lambda }}_{3}^{\alpha }-3\right],$$

with the isochoric principal stretches ${\bar{\lambda }}_{a}=J^{-1/3}\lambda_{a}$. The nonlinearity parameter α quantifies the strain stiffening behavior, as well as the compression‐tension asymmetry. If α was positive, the material response shows pronounced strain stiffening in tension, while a negative sign causes the opposite trend with more pronounced strain stiffening in compression. The absolute value then quantifies the asymmetric effect and the corresponding nonlinearity. The formulation Equation (3) is equivalent to the definition given by Ogden,^[^ ^49^ ^]^ but written in terms of the linear shear modulus μ. The volumetric contribution is defined as

(4) $$\Psi_{\mathrm{vol}}=\kappa \frac{1}{4}\left[J^{2}-1-2\ln J\right]$$

as described in Ref. [52], where the empirical coefficient β in the original formulation of Ogden^[^ ^53^ ^]^ is set to β = −2. the bulk modulus κ from the shear modulus μ was obtained using the relation

(5) $$\kappa =\mu \frac{2[1+\nu ]}{3[1-2\nu ]}$$

taken from the linear regime, where we set the Poisson's ratio ν to 0.45. To capture the geometrical boundary conditions of the experimental setup, the constitutive equations were implemented in a finite element (FE) model. It was assumed that the specimens fully adhere to the geometry and constrain the displacements at the contact surfaces to capture the inhomogenous deformations (**Figure** **3**). The model assumed perfect cylindrical or cuboidal geometries, thereby neglecting geometrical imperfections. Additionally, perfect bonding of the specimens was assumed to the testing device.

#### 2.7.2 Inverse Parameter Identification

To find the material parameters (μ, α) for which the model best fits the experimental results, the squared differences between the model predictions and the data was minimized, χ^2^, using nonlinear regression. In particular,

(6) $$\chi^{2}=\frac{\sum_{i=1}^{N}{\left[f_{i}^{exp}-f_{i}^{sim}\right]}^{2}}{\sum_{i=1}^{N}{\left[f_{i}^{exp}\right]}^{2}}$$

where N was the number of measured and simulated values. The scaling factor $\sum_{i=1}^{N}{\left(f_{i}^{exp}\right)}^{2}$, helps to tackle the problem of vanishing gradients of χ^2^ for small numerical values.^[^ ^54^ ^]^ To minimize χ^2^, the trust‐region‐reflective algorithm was used.^[^ ^55^ ^]^ Here, a parallelized version of the implementation in the Python package SciPy was adapted.^[^ ^56^ ^]^

## 3 Results

We first measure the mechanical response of pure basement membrane extracts (10 mgml^−1^ Matrigel or 14.6 mgml^−1^ Geltrex), pure collagen hydrogels (0.6; 1.2; 2.4 mgml^−1^), and blends of 1.2 mgml^−1^ collagen with Matrigel (1.2; 2.4; 3.6 mgml^−1^) under compression and tension.

Hydrogels containing collagen show an asymmetric mechanical response with higher forces in tension compared to compression (**Figure** **4**).

To quantify the mechanical behavior, we fit a finite element simulation, implementing the hyperelastic Ogden model, to the measured experimental response under compression and tension.

The model utilizes only two parameters, the shear modulus μ, characterizing the stiffness, and the nonlinearity parameter α, characterizing the strain‐stiffening and the compression‐tension asymmetry. The Ogden model is able to robustly capture the nonlinear compression‐tension asymmetry (**Figure** **5**a,b). Moreover, the model parameters obtained from compression‐tension experiments (Table 3) can predict the simple shear behavior of collagen 1.2 mgml^−1^ gels reasonably well (Figure 5c).

For increasing concentrations of pure collagen hydrogels, we observe increasing shear moduli μ (Figure 5b). Pure basement membrane extracts (10 mgml^−1^ Matrigel and 14.6 mgml^−1^ Geltrex) exhibit shear moduli comparable to pure 2.4 mgml^−1^ collagen gels, as do composite 1.2 mgml^−1^ collagen gels with higher Matrigel concentrations (2.4 or 3.6 mgml^−1^). Composite 1.2 mg/ml collagen gels with low Matrigel concentration (1.2 mgml^−1^) have a slightly lower stiffness, which, however, is still higher than that of pure 1.2 mgml^−1^ collagen.

Increasing the concentration of pure collagen results in higher absolute values for the nonlinearity parameters α, indicating a more pronounced strain‐stiffening in tension (Figure 5b). By contrast, Matrigel and Geltrex show small α values, indicating a nearly linear behavior in compression and tension for the measured strain range. Blends of 1.2 mgml^−1^ collagen with increasing concentrations of Matrigel show decreasing α values, indicating that higher Matrigel concentrations linearize the mechanical response of the material.

**Figure 6:**
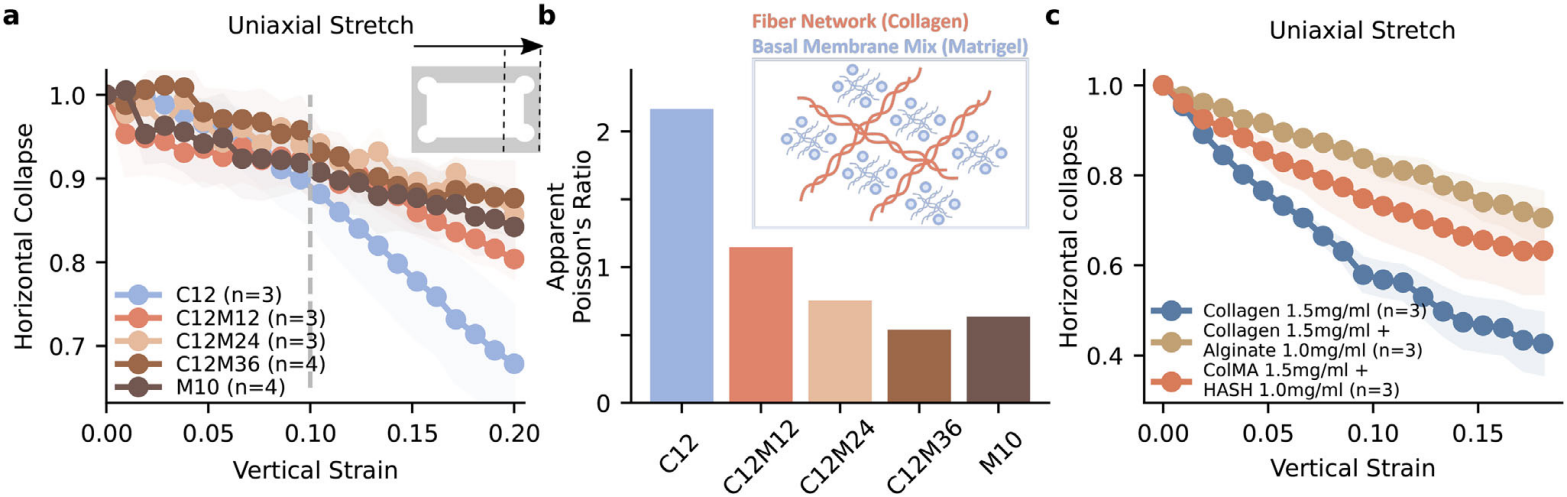
The addition of filler materials reduces the collapse of collagen fiber networks. a) Horizontal collapse of collagen and matrigel composite hydrogels measured under vertical stretch using uniaxial stretch experiments combined with brightfield microscopy. Solid lines and shaded areas indicate the mean±se, and n indicates the number of individual samples. b) The apparent Poisson's ratio derived from the slope of a linear fit to the data shown in (a) between a vertical strain of 0.1 and 0.2 (indicated by gray dashed line in (a). c) Horizontal collapse of collagen mixture gels measured for pure collagen hydrogels and collagen‐based hydrogels containing different filler materials. Collagen–alginate hybrid gels exhibit a low degree of chemical bonding, whereas methacrylated collagen with HA‐SH shows a higher degree of chemical bonding. Solid lines and shaded areas indicate the mean±se, and n indicates the number of individual samples.

A potential explanation for the more pronounced compression‐tension asymmetry of pure collagen hydrogels compared to composite hydrogels is that Matrigel may act as a filler material and thus partially prevent the collapse of the collagen fiber network. To test this hypothesis, we conduct uniaxial stretch tests, where the horizontal collapse in gel height is measured for increasing vertical strain up to 0.2. Above a vertical strain of 0.1, pure collagen networks exhibit a strong collapse in height, whereas pure Matrigel as well as Matrigel‐collagen blends collapse much less (**Figure** **6**a,b). To test whether the observed prevention of collagen fiber collapse is attributable to the volume‐preserving properties of Matrigel, we explore two additional blends: i) alginate‐collagen blends, which interact primarily through weak non‐covalent interactions, and ii) methacrylated collagen with thiol‐modified hyaluronic acid, which form covalent cross‐links. Both materials prevent collagen network collapse (Figure 6c), suggesting that volume preservation is a critical mechanism preventing fiber buckling.

**Figure 7:**
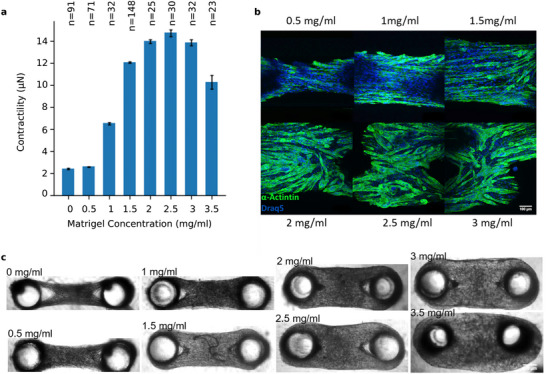
Matrigel‐collagen matrix composition alters the cellular organization and contractile forces of self‐assembled muscle tissues. a) Contractility of self‐assembled muscle tissues generated from 7500 skeletal muscle cells (C2C12) in different matrigel concentrations at a constant collagen concentration of 0.6 mgml^−1^. After 6 days in culture, the contractile forces are measured in response to electrical stimulation. Bars indicate mean±se and n indicates the number of individual microtissues. A maximum contractility is observed for microtissues at an intermediate Matrigel concentration of 2.5 mgml^−1^. b) Maximum intensity projected images of immunofluorescence stained microtissues (green: α‐actinin, blue: nuclei) with different Matrigel concentrations measured using confocal microscopy. Above a concentration of 1.5 mgml^−1^ Matrigel, the myotubes loose their proper alignment. The scalebar indicates 100 µm. c) Brightfield images of artificial muscle tissues for different Matrigel concentrations. The scalebar indicates 300 µm. Figure adapted from Ref. [43, 57].

To test if Matrigel‐collagen composites can also be used to tune cell behavior, we investigate self‐organized muscle tissues from C2C12 myoblasts cells with varying Matrigel‐collagen concentrations. As described in previous studies,^[^ ^25^, ^26^, ^43^, ^57^ ^]^ muscle tissues are assembled around two flexible pillars and differentiated over 6 days, after which the tissue contractility is measured under electrical stimulation as a metric for tissue function. It is known that skeletal muscle cells require the presence of laminins for proper differentiation into force‐generating myotubes.^[^ ^58^, ^59^ ^]^ In line with this, we observe that with increasing Matrigel concentration, the active (electrically stimulated) force generated by the tissue increases. The active force is largest for an intermediate Matrigel concentration of 2.5 mgml^−1^, but then decreases for higher Matrigel concentrations (**Figure** **7**a). This is explained by a decline in cell alignment in tissues at higher Matrigel concentration (Figure 7b), and by a decline in lateral tissue compaction (Figure 7c). Hence, even though the individual cells may still generate large active forces, the failure to align results in smaller end‐to‐end forces measured at the two pillars, which are insensitive to orthogonal forces within the tissue.

## 4 Discussion

In this work, we investigate the mechanical behavior of pure collagen hydrogels, pure basement membrane extracts (Matrigel or Geltrex), and Matrigel‐collagen blends. We quantify the nonlinear mechanical properties and the compression‐tension asymmetry using the hyperelastic Ogden model, which has two material parameters, the shear modulus μ, and the nonlinearity parameter α. This constitutive material model is incorporated into a finite element (FE) model to account for the inhomogeneous deformation states during mechanical testing due to the boundary conditions. The applicability of the Ogden model and its implementation in an FE simulation is verified by predicting the material response for simple shear from compression‐tension data (Figure 5c).

Typically, we test our samples first under shear and subsequently under tension and compression. This causes a potential preconditioning effect on the compression‐tension behavior from the previous loading mode. Therefore, our compressive and tensile measurements should be considered a lower estimate, as unconditioned samples may achieve higher stiffness values. Indeed, when we switch the order of the tested loading modes (Figure S11, Supporting Information), the stiffness values derived from the tension/compression data are higher.

In this work, we ignore any time‐dependent visco‐elastic effects, although in particular collagen hydrogels show pronounced hysteresis during cyclic loading (Figure 2a) and stress relaxation (Figure S12, Supporting Information). However, for small strains, both collagen and Matrigel show predominantly elastic behavior, as indicated by loss modulus values that are one order of magnitude below the storage modulus (Figure S13, Supporting Information).

Also of note, collagen network formation is strongly affected by the polymerization temperature. Lower temperatures are known to result in fiber networks with increased pore sizes and higher stiffness values^[^ ^15^, ^60^, ^61^, ^62^ ^]^), which is consistent with our observations (Figures S8 and S10, Supporting Information). Unless noted otherwise, we keep the temperature constant for each measurement setup by heating the rheometer plates or the medium to 37°C, to allow for direct comparison.

Despite these technical challenges, the stiffness values of our pure collagen hydrogels reported here are in agreement with previous studies.^[^ ^20^, ^63^, ^64^, ^65^ ^]^ Also, the stiffness of pure Matrigel hydrogels is in the range of previously reported values. For pure Matrigel (10 mgml^−1^), we obtain a shear modulus of 132 Pa, which is in good agreement with storage modulus values in the range of 80–100 Pa measured in previous studies^[^ ^66^, ^67^ ^]^ (at 0.5 –1 Hz) for similar concentrations (Figure 2; Figure S13, Supporting Information). Geltrex, another commercially available basement membrane mixture (both derived from murine Engelbreth‐Holm‐Swarms (EHS) tumors), behaves similarly to Matrigel. Previous studies have shown nonlinear effects for Matrigel at strains above 30–50%.^[^ ^67^, ^68^ ^]^ However, for the strain range investigated in the current study (up to 10% in compression/tension and 20% in shear), Matrigel behaves as a linear material.

For the mixed hydrogels, the stiffness is influenced by the ratio of Matrigel and collagen. The addition of Matrigel to 1.2 mgml^−1^ collagen gels leads to an initial increase in the shear modulus, as also observed by others.^[^ ^33^, ^69^, ^70^ ^]^ Further increasing the Matrigel concentration above 2.4  to 3.6 mgml^−1^ does not show an additional effect on the stiffness values.

Confocal reflection microscopy imaging shows that the collagen network remains intact when Matrigel is added, while the pore size increases slightly with increasing Matrigel concentration (Figures S9 and S10, Supporting Information). This is in line with previous studies.^[^ ^33^, ^69^ ^]^ The pore size of Matrigel, which is reported to be on the order of tens to hundreds of nanometers,^[^ ^71^, ^72^ ^]^ is below the resolution limit of our measurements.

Typically, collagen fiber networks exhibit pronounced fiber buckling under stretch, resulting in strong vertical collapse and abnormally high Poisson's ratio (>1). From stretch measurements under uniaxial tension, we obtain abnormal apparent Poisson's ratios for collagen of approximately 2, similar to previously reported values.^[^ ^73^, ^74^ ^]^ This vertical collapse of the individual fibers is the main mechanism for the pronounced compression‐tension asymmetry of collagen.^[^ ^15^, ^63^, ^75^ ^]^

Matrigel is not the only filling material that only minimally affects the polymerization of collagen, but greatly changes the bulk mechanical properties. A recent study^[^ ^76^ ^]^ reports that a collagen‐hyaluronic acid composite hydrogel also prevents embedded cells from compacting the matrix, similar to our finding in Matrigel‐collagen composite muscle tissues. In line with this, we observe similar behavior in collagen‐alginate composite hydrogels with low chemical bonding as well as in methacrylated collagen hydrogels with high chemical bonding, suggesting that the volume preserving properties of the filler material cause the observed reduction of the network collapse. These results are also in agreement with reports that the addition of cells or incompressible spheres to collagen hydrogels prevents the collapse of the collagen fibers under compression and reduces the compression‐tension asymmetry, similar to the behavior of real tissues.^[^ ^11^ ^]^

In summary, our main finding is that the compression‐tension asymmetry of collagen hydrogels, as expressed by the α parameter of the Ogden model, decreases with the addition of Matrigel. This can be explained by considering Matrigel as a filler material between collagen pores, preventing a collapse of the fiber network under compression. Indeed, our uniaxial stretch experiments show that hydrogel composites of collagen type I and filler materials Matrigel, Alginate, or thiol‐modified hyaluronic acid collapse much less compared to pure collagen gels (Figure 6). This aligns with the observed decrease in lateral tissue compaction and cellular organization in self‐assembled microtissues at higher Matrigel concentrations (Figure 7), showing that filler material concentration can be used to tune cell behavior.

## Conflict of Interest

The authors declare no conflict of interest.

## Author Contributions

D.B., J.H., B.F., and S.B. contributed equally to this work. S.B., B.F., and P.S. conceptualized the study and acquired funding. B.F., S.B., G.H., and S.S. supervised the study. B.F., S.B., J.H., and D.B. wrote the initial manuscript draft. D.B. conducted mechanical experiments. D.B. and R.G. conducted uniaxial stretch experiments. M.S. and S.W. conducted and evaluated the muscle microtissue experiments. J.H. implemented the simulation code and performed the numerical studies. J.H. and D.B. analyzed the data and visualized the results. All authors discussed the results, reviewed, and edited the manuscript.

## Supporting information

Supporting Information

## Data Availability

The data that support the findings of this study are available from the corresponding author upon reasonable request.
