# Supplementary material for: Compression‐Tension‐Asymmetry and Stiffness Nonlinearity of Collagen‐Matrigel Composite Hydrogels
Source: Adv Healthc Mater. 2025 Dec 5;15(9):e03052. doi: 10.1002/adhm.202503052 (PMC12973349; doi:10.1002/adhm.202503052)
Supplement: Supplementary file 1 — Supporting Information [file ADHM-15-0-s001.pdf]

# 7 Supplementary Information

| mixture | $\alpha\,[-]$ | $\mu\,[Pa]$ |
|---|---|---|
| C06 | 69.37 | 42.95 |
| C12 | 79.24 | 77.18 |
| C24 | 93.91 | 119.64 |
| C12M12 | 80.61 | 102.48 |
| C12M24 | 78.00 | 124.14 |
| C12M36 | 56.88 | 122.55 |
| Matri | -4.00 | 132.21 |
| Geltrex | 18.21 | 119.80 |

Table 3: Material parameter values for the one-term Ogden model obtained by an inverse analysis. The model is fitted to the mean response in compression and tension measured on the rheometer setup

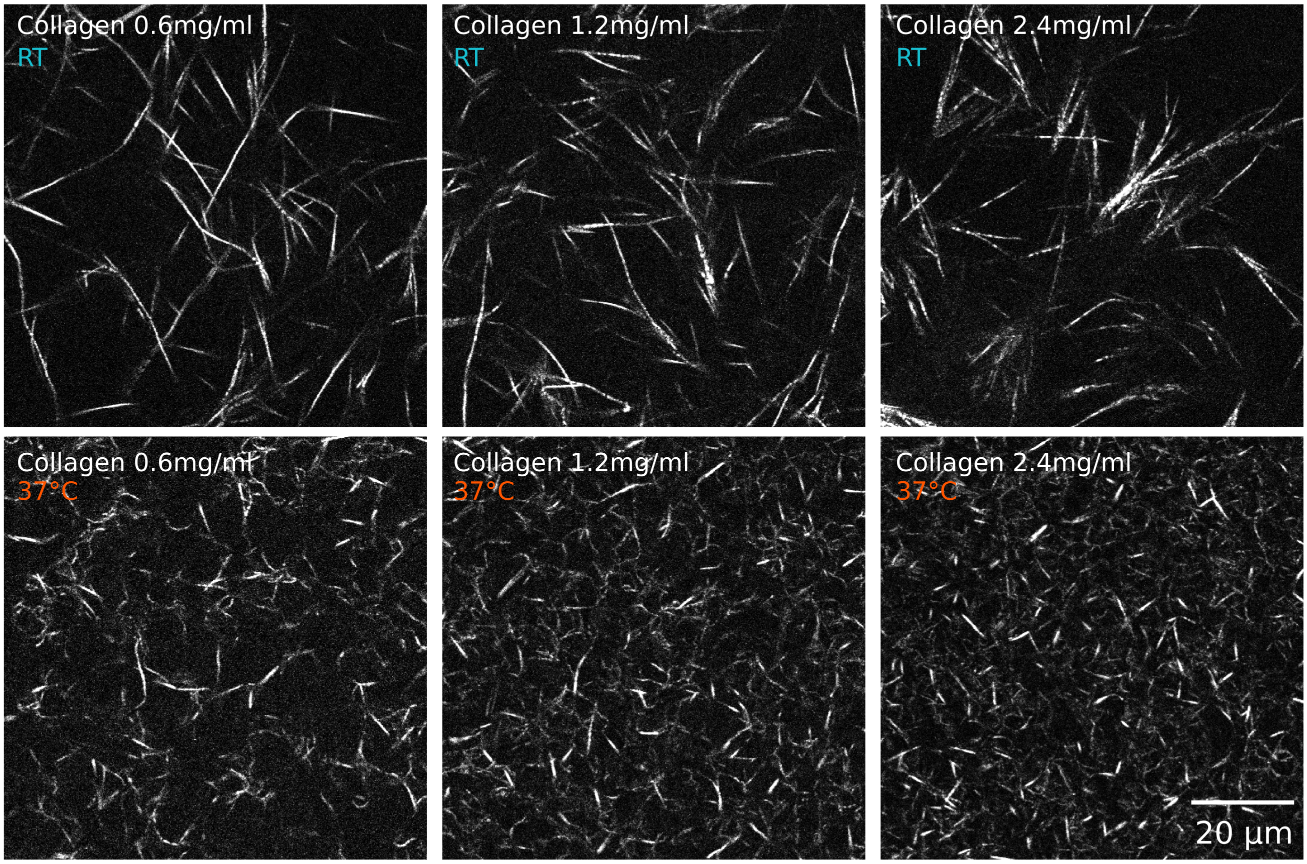


Figure 8: **Microstructure of collagen type I hydrogels.**, Confocal reflection microscopy images showing the collagen fiber networks at different polymerization temperatures (columns) and collagen concentrations (rows). For an increasing collagen concentration, we observe a decrease in pore size in agreement with previously studies [60, 42]. Collagen gels polymerized at room temperature (RT) exhibit a increased pore size and thicker fibers compared to those polymerized at 37°[60].

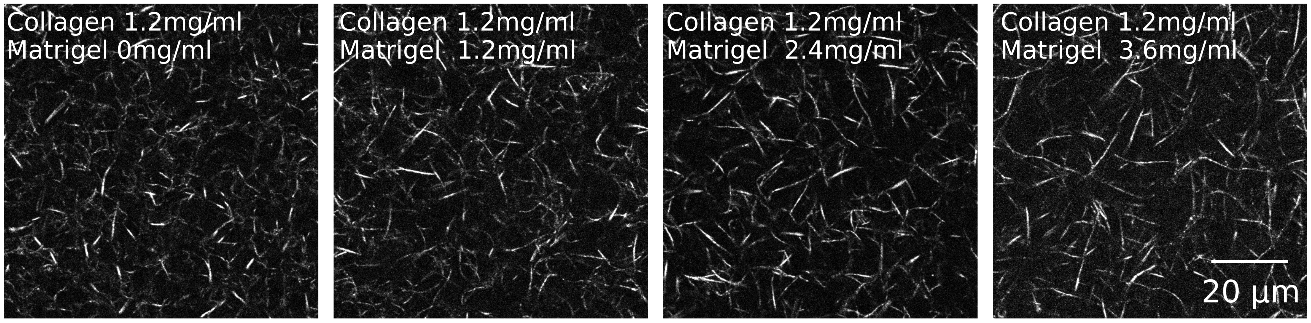


Figure 9: **Microstructure of hydrogels for varying ratios of Matrigel and collagen type I**, Confocal reflection microscopy images showing the collagen fiber networks (1.2 mg/ml) that are co-polymerized with different concentrations of Matrigel at 37°C. We observe larger pore sizes with increasing Matrigel concentrations.

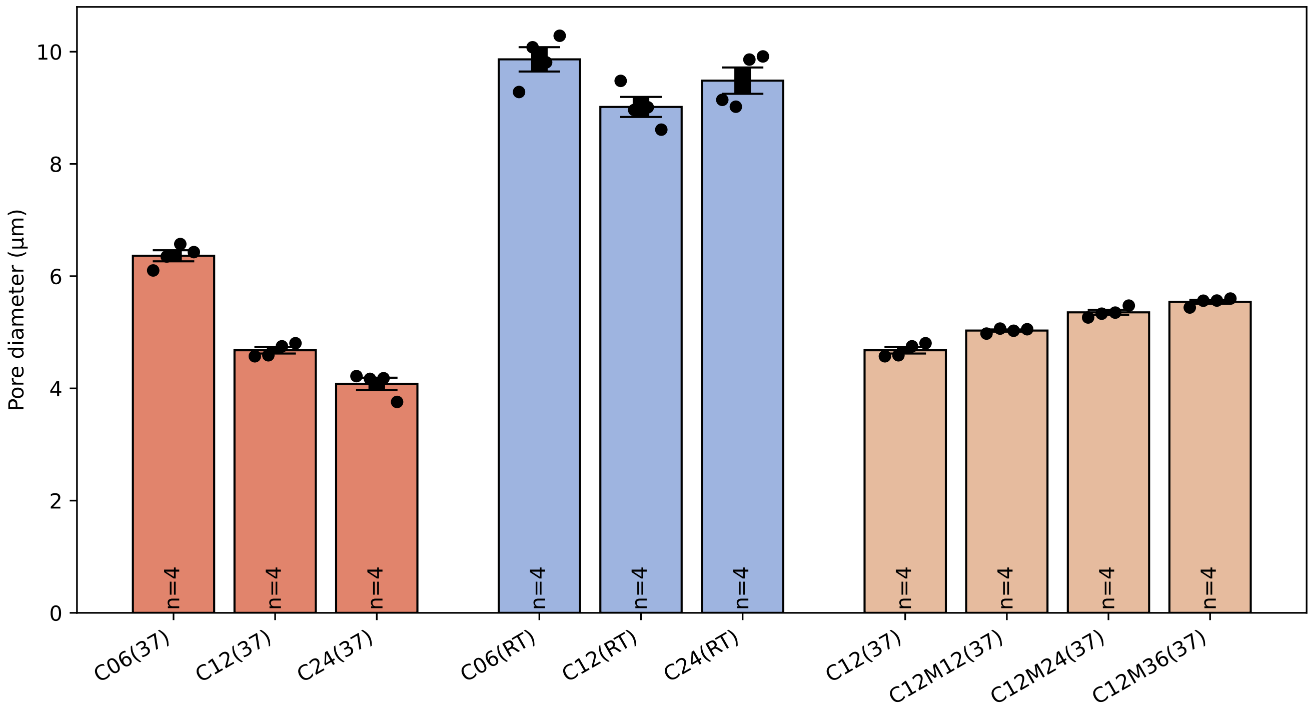


Figure 10: **Pore diameters in Matrigel-collagen composite hydrogels**, 3D pore diameters are estimated from 2D confocal reflection microscopy images (SI Figure 8, 9), similar as described in [39, 40]. Pore diameters are calculated for collagen gels at a concentration of 0.6 mg/ml (C06), 1.2 mg/ml (C12), 2.4 mg/ml (C24), and Matrigel-collagen blends all using 1.2 mg/ml collagen and 1.2 mg/ml Matrigel (C12M12), 2.4 mg/ml Matrigel (C12M24), and 3.6 mg/ml Matrigel (C12M36). RT indicates collagen gels polymerized at room temperature, and 37 indicates gels polymerized at 37°C. Bars indicate mean±se and n indicates the number of individual hydrogels. Overall, we observe that pore diameters decrease with increasing collagen concentration. C24 at room temperature is an outlier in this trend, which is likely to be caused by artifacts due to fiber bundling observed in SI Figure 8. Increasing the temperature from room temperature to 37°C leads to a decrease in pore diameters for all collagen concentrations. For increasing Matrigel concentrations at a temperature of 37°C, we observe a slight increase in pore diameters.

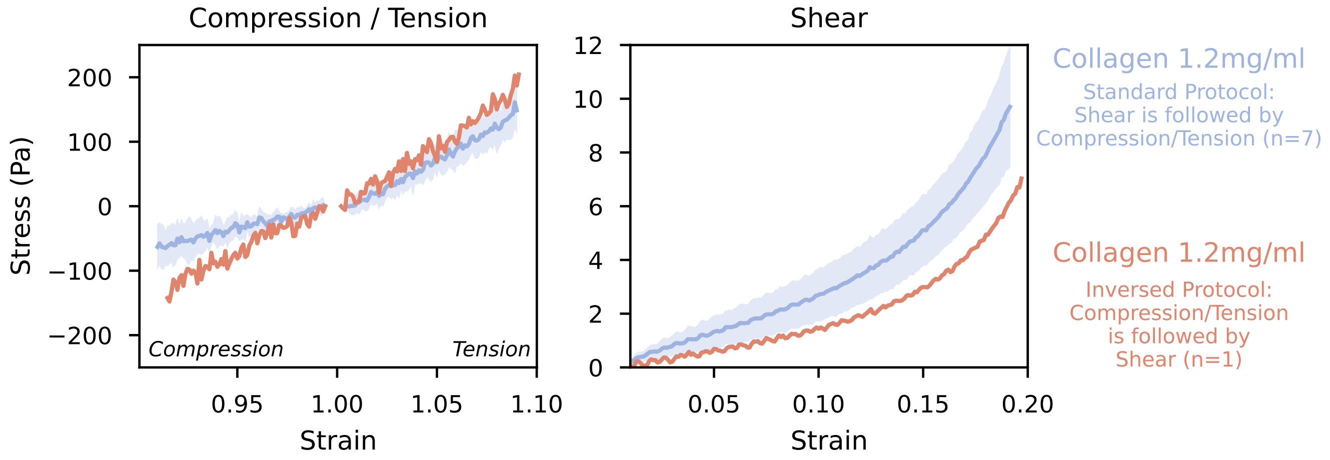


Figure 11: **Inversed order for compression-tension and shear tests**, Stress-strain relationship of 1.2 mg/ml collagen hydrogels under compression and tension (left) and under shear (right), both obtained from rheometer experiments. The blue curve shows the standard protocol where shear tests are performed first, followed by compression and tensile tests. The orange curve shows the inversed protocol order, where the compression and tension tests are performed before the shear test. Lines and shaded areas show the mean and standard deviation over individual specimens. The curves show the average of the first loading and unloading cycle, and n indicates the number of specimens measured.

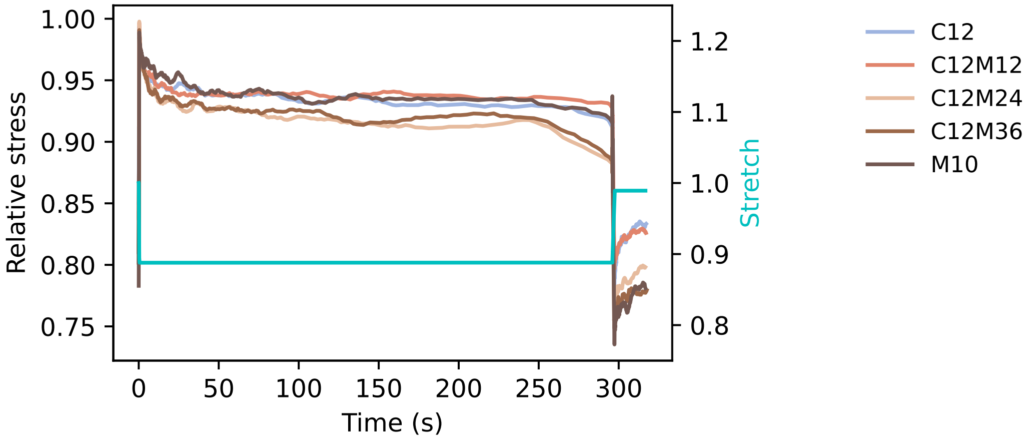


Figure 12: **Compressive stress relaxation tests**, Time-dependent relaxation of Matrigel-collagen composite hydrogels after compression step up to 10 % strain (green) measured using the rheometer setup. Lines show the relative stress normalized by the respective peak stress averaged over all individual samples (n) per hydrogel composition. The tested hydrogels are collagen gels at a concentration of 1.2 mg/ml (C12, n=7), Matrigel at a concentration of 10 mg/ml (n=3), and Matrigel-collagen blends all using 1.2 mg/ml collagen and 1.2 mg/ml Matrigel (C12M12, n=3), 2.4 mg/ml Matrigel (C12M24, n=3), or 3.6 mg/ml Matrigel (C12M36, n=3).

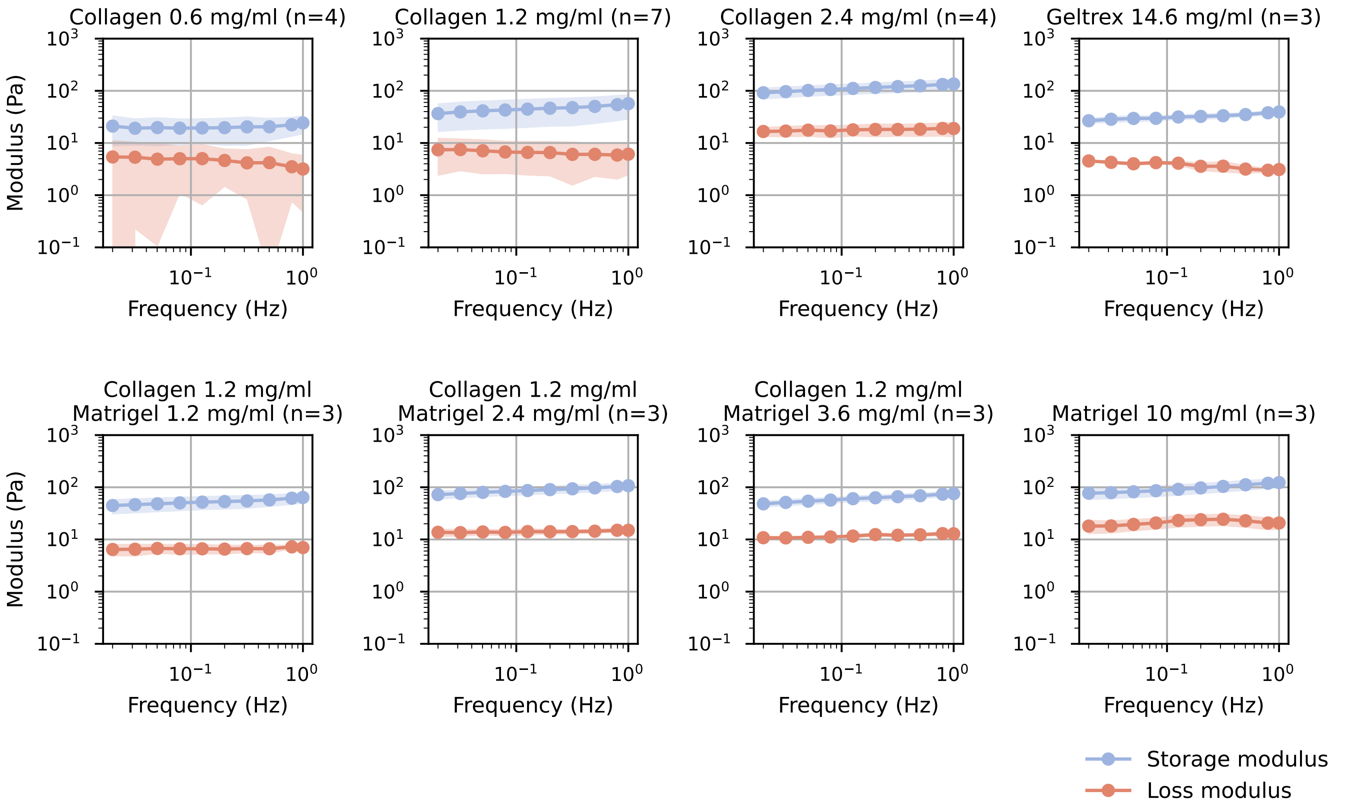


Figure 13: **Frequency dependent response of hydrogel mixtures**, Storage modulus (blue) and loss modulus (orange) derived from oscillatory frequency sweeps (0.02-1 Hz) with a plate-plate rheometer at a strain amplitude of 0.01. Hydrogels consist of Matrigel, Collagen and Geltrex at different concentrations. Lines indicate mean values and the shaded areas visualize the standard deviation. n denotes the number of individual samples.
